# Insulin-response epigenetic activation of *Egr-1* and *JunB* genes at the nuclear periphery by A-type lamin-associated pY19-Caveolin-2 in the inner nuclear membrane

**DOI:** 10.1093/nar/gkv181

**Published:** 2015-03-09

**Authors:** Kyuho Jeong, Hayeong Kwon, Jaewoong Lee, Donghwan Jang, Yunbae Pak

**Affiliations:** Department of Biochemistry, Division of Applied Life Science (BK21 Plus Program), PMBBRC, Gyeongsang National University, Jinju 660-701, Korea

## Abstract

Insulin controls transcription to sustain its physiologic effects for the organism to adapt to environmental changes added to genetic predisposition. Nevertheless, insulin-induced transcriptional regulation by epigenetic factors and in defined nuclear territory remains elusive. Here we show that inner nuclear membrane (INM)-integrated caveolin-2 (Cav-2) regulates insulin-response epigenetic activation of *Egr-1* and *JunB* genes at the nuclear periphery. INM-targeted pY19-Cav-2 in response to insulin associates specifically with the A-type lamin, disengages the repressed *Egr-1* and *JunB* promoters from lamin A/C through disassembly of H3K9me3, and facilitates assembly of H3K9ac, H3K18ac and H3K27ac by recruitment of GCN5 and p300 and the subsequent enrichment of RNA polymerase II (Pol II) on the promoters at the nuclear periphery. Our findings show that Cav-2 is an epigenetic regulator of histone H3 modifications, and provide novel mechanisms of insulin-response epigenetic activation at the nuclear periphery.

## INTRODUCTION

Insulin exerts its physiologic effects through the regulation of gene expression (1). As many as 150 genes are reported to be regulated by insulin in various tissues and cell lines (2). A microarray profiling of human skeletal muscle has shown that the expression of ∼800 genes is affected during a hyperinsulinemic clamp (3). Nonetheless, the mechanisms of insulin-response transcriptional regulation unraveling stepwise dynamics of the DNA–protein interactions and posttranscriptional modification of proteins in the transcription complex, and changes in the modification of histones associated with insulin-response genes remain elusive. Moreover, the spatial epigenetic repression and activation of insulin-response genes in the specific nuclear territory are unknown, and the epigenetic factors involved in the transcriptional regulation have yet to be identified.

Caveolins, lipid rafts-associated integral membrane proteins, are required for a variety of cell signaling and mechanotransduction (4,5). Relative to the well-characterized Cav-1 and Cav-3, the functional role of Cav-2 has remained poorly characterized. pY19-Cav-2 is transported to the inner nuclear membrane (INM) and associates with lamin A/C in response to insulin (6,7). The INM-integrated pY19-Cav-2 decreases histone H3 lysine 9 trimethylation (H3K9me3), increases transcriptional activation of Elk-1 and signal transducer and activator of transcription 3 (STAT3) (6), and promotes induction of insulin-response *Egr-1* and *JunB* genes (8). *Egr-1* has been reported to be induced by mitogens and differentiation stimuli including insulin and related to maintain sensitivity to insulin (9–11). Loss of *Egr-1* function in insulin-resistant adipocytes restores activity of insulin receptor substrate-1 and increases insulin sensitivity (12). As a transcription factor, Egr-1 regulates expression of *TNF-α*, *PTEN* and *SOCS-1* (13–15) and exerts its inhibitory effect on adipocyte differentiation in 3T3L1 cells (16). Thus, the regulation of *Egr-1* expression is important for cellular differentiation and mitogenesis of insulin signaling. However, epigenetic regulatory mechanisms involved in histone modifications and RNA Pol II transcription which control *Egr-1* induction are unknown.

Nuclear lamina, a network of lamins associated with integral and peripheral INM proteins at the nuclear periphery, mediates interaction with chromatin (17,18). The lamina association with chromatin is thought to be through interaction of lamins with histones and DNA (18–21) and has been proposed to occur via lamin association domains including mostly repressed genes (22–24). Thus, heterochromatin interaction with nuclear lamina has been suggested to be a mechanism for maintaining silent chromatin at the nuclear periphery (25,26). Nuclear lamins consisting of the A-type lamins, lamin A and C, and the B-type lamins, lamin B1 and B2 (27) play important roles in spatial organization of chromosome territories and function in the regulation of replication, transcription, and epigenetic modifications of chromatin (28–30). Although it has been shown that the A- and B-type lamins form their distinctive domains at the nuclear periphery and provide a different environment for the regulation of gene expression (29), the molecular mechanisms of epigenetic regulation of chromatin via these domains and the identification of A- versus B-type lamin-associated epigenetic factors responsible for chromatin remodeling and RNA Pol II transcription remain largely unexplored.

We explored whether the A-type lamin-associated Cav-2 localized in the INM functions as an epigenetic regulator modulating histone H3 modifications for the activation of insulin-response genes. In this study, we show that lamin A/C-associated pY19-Cav-2 facilitates insulin-response epigenetic activation of *Egr-1* and *JunB* genes at the nuclear periphery via dissociation of the inactivated *Egr-1* and *JunB* promoters from lamin A/C through disassembly of H3K9me3 on the promoters and recruitment of specific histone acetyltransferases and subsequent sequential enrichment of the corresponding histone H3 acetylations and RNA Pol II on the target gene promoters. Thus, we identify Cav-2 in the INM as an insulin-response epigenetic regulator of histone H3 modifications and novel mechanisms by which Cav-2 manifests insulin-response epigenetic activation at the nuclear periphery through its phosphorylation and association with A-type lamin.

## MATERIALS AND METHODS

### Materials

Cell culture reagents were purchased from Gibco. Antibodies and reagents used were as follows: Cav-2 (BD 610685), lamin A/C (BD 612162) and E-cadherin (BD 610182), antibodies from BD Transduction Laboratories; lamin A/C (sc-20681), lamin B1 (sc-30264), α-tubulin (sc-5286), maltose-binding protein (MBP) (sc-73416), emerin (sc-15378), GFP (sc-9996), histone H3 (sc-10890), histone H1 (sc-8030), protein-tyrosine phosphatase 1B (PTP1B) (sc-1718), GCN5 (sc-20698) and p300 (sc-585) antibodies from Santa Cruz Biotechnology; pY19-Cav-2 (ab3417), lamin B-receptor (LBR) (ab169306), H3K9me3 (ab8898), H3K9ac (ab32129), H3K18ac (ab1191) and H3K27ac (ab4729) antibodies from Abcam; GFP (#2555) antibody from Cell Signaling; AcH3 (06-599), RNA Pol II (05-623), H3K4ac (07-539), and H3K14ac (07-353) antibodies from Millipore; FITC-conjugated anti-mouse (F9006), TRITC-conjugated anti-rabbit (T5268), horseradish peroxidase (HRP)-conjugated anti-mouse (A4416) and anti-rabbit (A6154) antibodies and 4′-6-diamidino-2-phenylindole (DAPI) (D8417), curcumin (C1386), butyrolactone 3 (M2449) and sodium *ortho*-vanadate (S6508) from Sigma; human insulin from Eli Lilly.

### Cell culture

Human insulin receptor-expressed rat 1 fibroblast (Hirc-B) cells expressing Cav-2 and Cav-2 short-hairpin RNA (shRNA) stable Hirc-B cells (31–33) were grown in Dulbecco's modiﬁed Eagle's medium (DMEM) containing 5 mM d-glucose supplemented with 10% (v/v) fetal bovine serum (FBS) (Sigma), 100 nM methotrexate, and 0.5% gentamycin in a 5% CO_2_ incubator at 37°C. Human embryonic kidney (HEK) 293T cells expressing no Caveolins (7,34–35) were grown in DMEM containing 5 mM d-glucose supplemented with 10% (v/v) FBS and 0.5% penicillin/streptomycin in a 5% CO_2_ incubator at 37°C.

### Plasmids

A full-length Cav-2 cDNA (GenBank accession no. NM_131914) was subcloned into pcDNA3 vector (Invitrogen) as described (36,37). Cav-2 oligomerization domain deletion (Δ47–86-Cav-2) and tyrosine phosphorylation deficient (Y19A-Cav-2) mutants were generated by polymerase chain reaction (PCR) mutagenesis from the wild type (WT) pcDNA-Cav-2. The resulting entry vectors of WT and mutants were converted into self-constructed GFP tagging destination expression vector (pEGFP-N1 vector, Clontech Laboratories). The Cav-2 and Δ47–86-Cav-2 cloned into pEGFP-N1 vector were point-mutated at codon 203 from threonine to histidine in the GFP sequence of pEGFP-N1 vector as described (6) by PCR mutagenesis to generate Cav-2-PAGFP and Δ47–86-Cav-2-PAGFP for photoactivation. Cav-2 domain truncation mutants including Δ1–13-Cav-2, Δ1–46-Cav-2, Δ1–70-Cav-2, Δ47–86-Cav-2 and Δ120–162-Cav-2 were generated by using the WT Cav-2-GFP (residues 1–162) as template via EZchange site-directed mutagenesis kit (Enzynomics). The resulting entry vectors of WT and mutants were converted into self-constructed MBP tagging destination expression vector (pMGWA vector). All expression vectors were verified by sequencing.

### Duolink *in situ* proximity ligation assay

*In situ* proximity ligation assay (PLA) was performed using the Duolink Detection Kit (Sigma) according to the instructions. Hirc-B cells were fixed, permeabilized and blocked as described (6), and then incubated with anti-Cav-2 and anti-lamin A/C, anti-pY19-Cav-2 and anti-lamin A/C, anti-Cav-2 and anti-lamin B1, anti-lamin A/C and anti-emerin, or anti-lamin B1 and anti-LBR antibodies at 4°C overnight. After washing, the oligonucleotide (minus and plus)-conjugated secondary antibodies were incubated for 1 h at 37°C. Subsequently, cells were washed and incubated with ligation solution for 30 min at 37°C. Finally, the ligated nucleotide circles were amplified by polymerase via rolling-circle amplification (RCA), and the RCA products were visualized by hybridization with fluorescence labeled oligonucleotides. Nuclei were fluorescently labeled with DAPI. The red fluorescent images were obtained using confocal microscope (IX-81; Olympus) with an attached 543 nm laser using a PlanApo 60×/1.40 oil immersion objective lens (Olympus).

### Nuclear fractionation

As described (7,38), cells were scraped with hypotonic lysis buffer (10 mM Tris–HCl, pH 7.5, 10 mM NaCl, 3 mM MgCl_2_, 1 mM sodium *ortho*-vanadate, 5 μg/ml aprotinin, 3 μg/ml pepstatin, 5 μg/ml leupeptin, 1 mM ethylenediaminetetraacetic acid (EDTA) and 1 mM dithiothreitol (DTT)) and homogenized using 10 strokes of a Dounce homogenizer and then centrifuged at 1000 rpm for 3 min at 4°C. The crude nuclear pellet was resuspended in nuclear isolation buffer (0.5% Nonidet P-40, 10 mM Tris–HCl, pH 7.5, 10 mM NaCl, 3 mM MgCl_2_, 1 mM sodium *ortho*-vanadate, 5 μg/ml aprotinin, 3 μg/ml pepstatin, 5 μg/ml leupeptin, 1 mM EDTA and 1 mM DTT) and incubated on ice for 5 min, and the nuclei were pelleted by centrifugation at 1000 rpm for 3 min at 4°C to yield the nuclear fraction. The initial supernatant was centrifuged at 12 500 × *g* for 15 min at 4°C to yield the crude cytoplasmic fraction and the membrane pellet comprising mitochondria, endoplasmic reticulum and plasma membrane. The crude cytoplasmic fraction was centrifuged at 100 000 × *g* for 60 min at 4°C using a TLA 100.3 rotor (Beckman) to yield the pure cytoplasmic fraction. The purified nuclei and the membrane pellet were incubated with lysis buffer A (2% Triton X-100, 20 mM Tris–HCl, pH 7.5, 280 mM NaCl, 10 mM NaF, 1 mM sodium *ortho*-vanadate, 5 μg/ml aprotinin, 3 μg/ml pepstatin, 5 μg/ml leupeptin, 1 mM EDTA and 1 mM DTT) containing 8M urea for 30 min at room temperature and lysis buffer B (1% Triton X-100, 150 mM NaCl, 10 mM Tris–HCl (pH 7.4), 1 mM EDTA, 1 mM ethylene glycol tetraacetic acid (EGTA) (pH 8.0), 0.2 mM sodium *ortho*-vanadate, 0.2 mM phenylmethylsulfonyl fluoride (PMSF), 0.5% Nonidet P-40 and 60 mM *n*-octylglucoside) for 30 min at 4°C, respectively. The lysates were centrifuged at 12 000 rpm for 10 min at 4°C and the supernatants were collected as nuclear lysates and total membrane lysates, respectively for immunoblot and immunoprecipitation analyses and *in vitro* binding assay.

### *In vitro* binding assay

The nuclear lysates (1 mg proteins) lysed with lysis buffer A containing 8M urea to fully solubilize lamins (see Supplementary Figure S1) were incubated with purified MBP-Cav-2, MBP-Δ1–13-Cav-2, MBP-Δ1–46-Cav-2, MBP-Δ1–70-Cav-2, MBP-Δ47–86-Cav-2, or MBP-Δ120–162-Cav-2 (100 μg) prebound to amylose resin at 4°C for 4 h. Amylose resins were washed four times with immunoprecipitation buffer (1% Triton X-100, 150 mM NaCl, 10 mM Tris–HCl (pH 7.4), 1 mM EDTA, 1 mM ethylene glycol tetraacetic acid (pH 8.0), 0.2 mM sodium ortho-vanadate, 0.2 mM PMSF, and 0.5% Nonidet P-40). Bound proteins were analyzed by immunoblotting.

### Photoactivation

**Figure 1.**
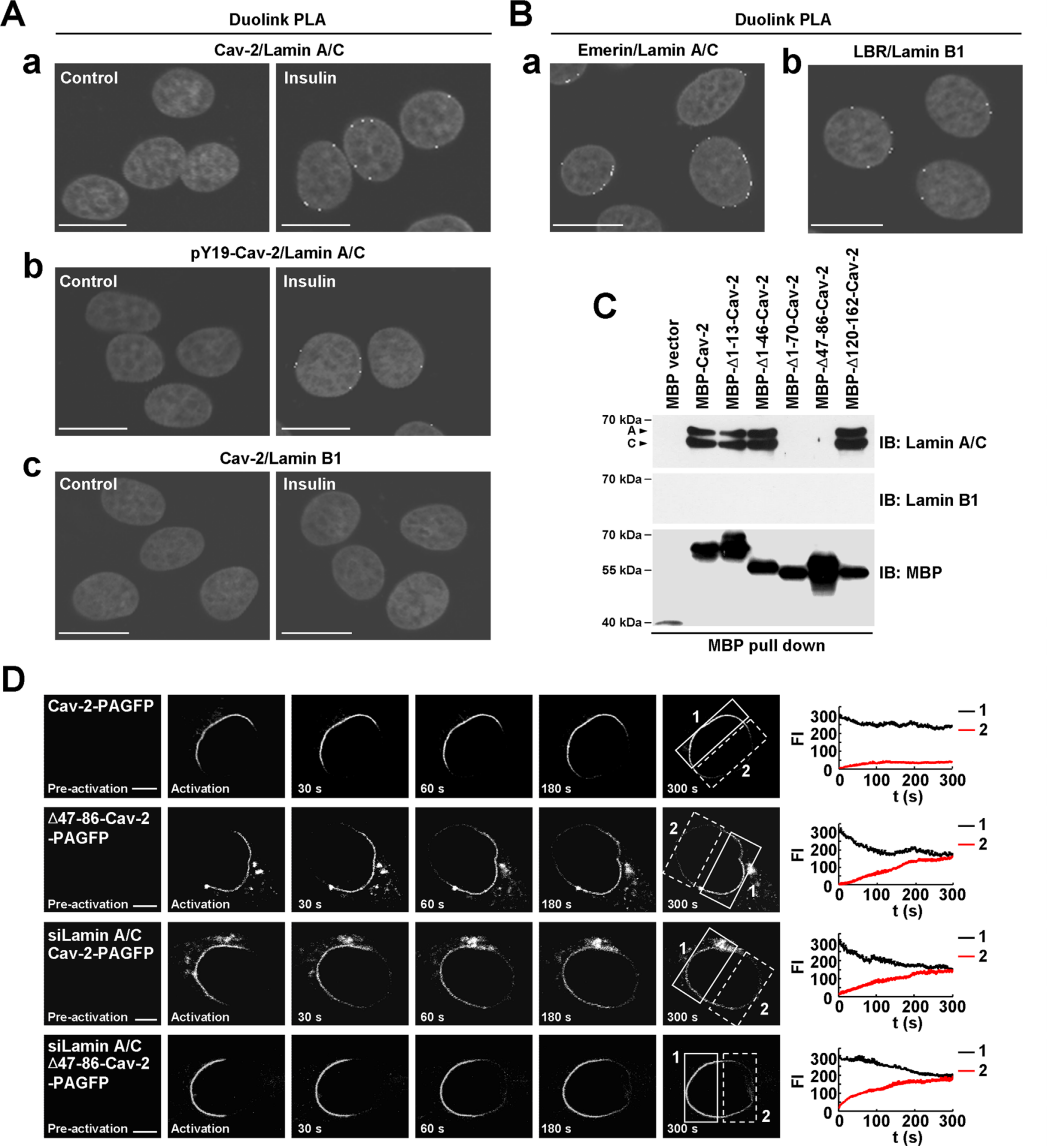
pY19-Cav-2 in the INM directly interacts with lamin A/C at the nuclear periphery in response to insulin. (**A** and **B**) Cav-2 interaction with lamin A/C but not lamin B1 in endogenous Cav-2-expressing Hirc-B cells. Insulin-induced interaction between Cav-2 and lamin A/C (**A, a**), pY19-Cav-2 and lamin A/C (**A**, **b**), or Cav-2 and lamin B1 (**A**, **c**) at the nuclear periphery was determined by Duolink PLA in Hirc-B cells treated with or without 100 nM insulin for 30 min. The *in situ* interactions between lamin A/C and emerin (**B**, **a**) and between lamin B1 and LBR (**B**, **b**) in Hirc-B cells were visualized by Duolink PLA. White dots in the gray-scale images represent the clusters of protein–protein interactions. Scale bars, 20 μm. (**C**) The binding motif of Cav-2 (residues 47–70) to lamin A/C. Nuclear lysates from HEK293T cells expressing no endogenous Caveolins were subjected to *in vitro* binding assay using MBP vector, MBP-Cav-2, MBP-Δ1–13-Cav-2, MBP-Δ1–46-Cav-2, MBP-Δ1–70-Cav-2, MBP-Δ47–86-Cav-2 or MBP-Δ120–162-Cav-2. (**D**) Association of Cav-2 in the INM with lamin A/C. HEK293T cells expressed with Cav-2-PAGFP or Δ47–86-Cav-2-PAGFP, or co-transfected with Cav-2-PAGFP or Δ47–86-Cav-2-PAGFP and siLamin A/C were subjected to photoactivation. The graph shows FI of Cav-2-PAGFP or Δ47–86-Cav-2-PAGFP in the NE region before (1: solid box) and after (2: dotted box) photoactivation (mean ± SE, *n* = 5). Scale bars, 5 μm.

HEK293T cells were plated on a 35 mm glass-bottom dish and transfected with Cav-2-PAGFP or Δ47–86-Cav-2-PAGFP, or co-transfected with siLamin A/C and Cav-2-PAGFP or Δ47–86-Cav-2-PAGFP for 48 h. Photoactivation of the Cav-2-PAGFP or Δ47–86-Cav-2-PAGFP was performed as described (6) on confocal microscope (IX-81; Olympus) with an attached 405 nm laser using a PlanApo 100×/1.35 oil immersion objective lens (Olympus). The nuclear envelope (NE) region was photoactivated at 10% laser power for 0.5 s, and GFP fluorescence was imaged every 3 s for 250 scans. Cav-2-PAGFP or Δ47–86-Cav-2-PAGFP fluorescence intensity (FI) in the same-sized boxed areas in the NE (see Figure 1D) was automatically quantified at each time point, background subtracted, and corrected for loss or gain of fluorescence during imaging by using FRAP Profiler Plugin of MBF-ImageJ. The FI was graphically depicted using Microsoft Excel. The mobility rate (%) of Cav-2-PAGFP or Δ47–86-Cav-2-PAGFP from the solid box (photoactivated area at *t*_0_ s) to the dotted box (mobile fluorescent signal measured area at *t*_300_ s) was quantified by *t*_300_ s FI/*t*_0_ s FI × 100.

### Silencing of lamin A/C and PTP1B by RNA interference

Small interfering RNA (siRNA) targets of lamin A/C and PTP1B were purchased from Bioneer Corp. (Daejon, Korea). The siRNA oligonucleotides were synthesized to the following target sequences: lamin A/C; sense (5′-CGUACGGCUCUCAUCAACU-3′) and antisense (5′-AGUUGAUGAGAGCCGUACG-3′), PTP1B; sense (5′-CGUCAGUCCCUUUGACCAU-3′) and antisense (5′-AUGGUCAAAGGGACUGACG-3′) and scramble control; 5′-GGAAAGACUGUUCCAAAAA-3′. The siRNAs were introduced into cells using DharmaFECT Transfection Reagents (Dharmacon) by following the procedure recommended by the manufacturer.

### Immunoprecipitation

The nuclear lysates lysed with lysis buffer A containing 8M urea were incubated with anti-Cav-2 or anti-GFP antibody by rotating overnight at 4°C and added with 30 μl of protein G or A plus Agarose (Calbiochem) and rotated further for 4 h at 4°C. The immunocomplexes were collected by centrifugation at 12 000 rpm for 10 min at 4°C and washed thrice in ice-cold immunoprecipitation buffer. After the final wash the pellet was resuspended in 20 μl of 2× sodium dodecyl sulfate-polyacrylamide gel electrophoresis (SDS-PAGE) sample buffer and analyzed by immunoblotting.

### Immunoblotting

The immunoprecipitates and the proteins from MBP pull-down and nuclear, membrane and cytoplasmic fractions were separated by SDS-PAGE and transferred to polyvinylidene difluoride membranes (Millipore). Membranes were blocked for 2 h in 2% non-fat dry milk in Tris-buffered saline-Tween (TBS-T) at room temperature and incubated overnight at 4°C in primary antibody in 2% bovine serum albumin (BSA) in TBS-T, followed by three washes in TBS-T. The membranes were incubated for 1 h at room temperature in HRP-conjugated secondary antibody in 2% non-fat dry milk in TBS-T and washed thrice in TBS-T. The membranes were developed using a Luminata™ Crescendo western HRP substrate (Millipore).

### Confocal microscopy and image analysis

Hirc-B cells were treated with or without 100 nM insulin for 30 min. The cells were fixed with 3.7% paraformaldehyde in phosphate-buffered saline (PBS) for 20 min and permeabilized with 0.1% Triton X-100 for 20 min at room temperature. Permeabilized cells were rinsed with PBS and blocked with 1% BSA in PBS for 1 h at room temperature. Cells were rinsed with PBS and incubated with anti-H3K9me3, anti-AcH3, anti-H3K9ac, anti-H3K18ac or anti-H3K4ac and anti-lamin A/C or anti-Cav-2 antibodies in 1% BSA in PBS for overnight at 4°C. After washing thrice with PBS, the cells were incubated with TRITC-conjugated anti-rabbit and FITC-conjugated anti-mouse antibodies in 1% BSA in PBS for 2 h at room temperature. Nuclei were fluorescently labeled with DAPI. Fluorescent images were obtained using an Olympus Fluoview 1000 confocal microscope attached to IX-81 inverted microscope equipped with PlanApo 60×/1.40 oil immersion objective lens (Olympus). FITC signals were excited using an Argon laser at 488 nm, TRITC signals using a He–Ne laser at 543 nm, and DAPI signals using Diode laser at 405 nm. FV10-ASW software (Olympus) was used to merge the images from FITC, TRITC and DAPI. Quantitation of co-localization of H3K9me3, AcH3, H3K9ac, H3K18ac or H3K4ac with lamin A/C was performed using the Colocalization Finder Plugin of ImageJ as described (6,7). The amount of co-localization of H3K9me3 or the acetylated histone H3 with lamin A/C, expressed as the percentage of pixels with threshold lamin A/C immunoreactivity that co-localized with threshold H3K9me3 or the acetylated histone H3 staining, was calculated as the number of co-localized pixels divided by the number of threshold pixels in the lamin A/C image. Averages and standard errors (SEs) were computed over five images per condition for a minimum of 20 cells per condition (*n* = 3).

### Ablation of endogenous Cav-2 by shRNA

Cav-2 shRNA stable Hirc-B cells were generated as described (33). Hirc-B cells were transfected with Cav-2 shRNA expressing plasmid (MISSION^®^ shRNA Bacterial Glycerol Stock, Sigma) for 48 h and subjected to incubation with 1 μg/ml puromycin (Clontech) to select for puromycin-resistant clones for a week. Independent colonies were then picked using cloning cylinder (Sigma), subcultured, and tested for Cav-2 expression by reverse transcription (RT)-PCR and immunoblot analysis. Stable cell lines that express a Cav-2 shRNA were then selected.

### RNA extraction and cDNA synthesis

Total RNA was extracted with TRIzol reagent (SolGent) according to the manufacturer's instructions. cDNA was generated using Accupower RT PreMix kit (Bioneer). The cDNA was used as the template for the subsequent real-time quantitative RT-PCR (qRT-PCR) analysis. The sequences of qRT-PCR primers are listed in Supplementary Table S1.

### Chromatin immunoprecipitation assay

Chromatin immunoprecipitation (ChIP) experiments were performed using the EZ ChIP™ chromatin IP kit (Millipore). Cells were fixed by 1% formaldehyde for 10 min at room temperature and quenched by 125 mM glycine for 5 min. Whole cell extracts were sonicated into 200- to 500-bp fragments for 30 × 20 s at 20% amplitude (Sonics Vibra Cell Sonicator) in lysis buffer C (1% SDS, 10 mM EDTA, 50 mM Tris–HCl, pH 8.1 and protease inhibitors), precleared using salmon sperm DNA, and then incubated with a specific antibody overnight at 4°C. For ChIP with anti-lamin A/C antibody, chromatin was prepared by lysis and sonication in lysis buffer D (10 mM Tris–HCl, pH 7.5, 10 mM KCl, 2 mM EDTA, 1% Triton X-100 and protease inhibitors) (39). After washing the immunoprecipitates, DNA/antibody complexes were eluted, crosslinks reversed and DNA was purified by QIAquick PCR purification kit (Qiagen). Input DNA starting from aliquots of sonicated sample was purified using QIAquick PCR purification kit. The purified DNA and input genomic DNA were analyzed by real-time quantitative PCR (qPCR) using SsoFastTM EvaGreen^®^ Supermix (Bio-Rad) and the Eco real time PCR system (Illumina). qPCR analysis of ChIP sample was normalized to input genomic DNA. For ChIP on the rat *Egr-1*, *JunB* or *Actb* gene, 14 pairs of PCR primers to cover from −10 to +10 kb of the transcription start sites (TSSs) of *Egr-1*, *JunB* or *Actb* gene were designed. The sequences of qPCR primers are listed in Supplementary Table S1.

### Fluorescence *in situ* hybridization

The DNA fragments for making hybridization probes were cloned into the pGEM-T easy vector system (Promega) from the cDNA of Hirc-B cells as follows: the *Egr-1* (1276 bp) used forward primer 5′-CTGACATCGCTCTGAATAACG-3′ and reverse primer 5′-CTAGGAGAAAAGGTTGCTGTC-3′. The *JunB* (1002 bp) used forward primer 5′-AAAATGGAACAGCCTTTCTAT-3′ and reverse primer 5′-TAGCAGCAACTGGCAGCCGTT-3′. Both fragments were nick-translated and labeled with red fluorescent dye Alexa Fluor 594 using the FISH Tag DNA kit (Invitrogen) according to the manufacturer's instructions. For immuno-fluorescence *in situ* hybridization (FISH), Hirc-B cells were treated with or without 100 nM insulin for 30 min. The cells were fixed in 3.7% paraformaldehyde, then permeabilized in 0.1% Triton X-100/PBS and incubated with anti-Cav-2 antibody at room temperature for 2 h and FITC-conjugated anti-mouse antibody for 1 h. After washing in PBS, cells were incubated with 1% formaldehyde for 5 min to fix antibodies prior to denaturing FISH steps. The hybridizations were performed using the FISH tag DNA kit (Invitrogen) according to the manufacturer's protocols. The *in situ* nuclear localization of *Egr-1* and *JunB* genes and Cav-2 localization in the INM were then analyzed using confocal microscope (IX-81; Olympus) with attached 543 and 488 nm lasers, respectively using a PlanApo 60×/1.40 oil immersion objective lens (Olympus).

### Statistics

Chemiluminescent images of immunoblots were analyzed by scanning densitometry using Kodak Gel Logic 100 Imaging System (Eastman Kodak Co.). Multiple exposure of each blot was used to obtain gray-scale images of each chemiluminescent band. Bands were visualized on a UV transilluminator and photographed. Data are expressed as mean ± SE. An unpaired Student's *t* test was used to compare treatment groups with significance established at a level of *P* < 0.05.

## RESULTS

### Direct interaction of INM-targeted pY19-Cav-2 with A-type lamin at the nuclear periphery in response to insulin

Having shown that INM-targeted pY19-Cav-2 from Golgi co-localizes and associates with lamin A/C in response to insulin (6,7), it was important to verify their direct interaction at the nuclear periphery in response to insulin and identify the binding motif of Cav-2 to lamin A/C. Duolink PLA confirmed insulin-induced interaction of Cav-2 and pY19-Cav-2 with lamin A/C at the nuclear periphery (Figure 1A, a and b) and showed no interaction between Cav-2 and lamin B1 (Figure 1A, c) in Hirc-B cells. *In situ* interactions of lamin A/C with emerin and of lamin B1 with LBR at the nuclear periphery are also demonstrated by the Duolink PLA (Figure 1B, a and b). To identify the lamin A/C-binding region of Cav-2, *in vitro* binding assay were performed using domain truncation mutants of Cav-2 with nuclear lysates of HEK293T cells. Lamin A/C interacted with WT Cav-2 (residues 1–162) and Δ1–13-Cav-2, Δ1–46-Cav-2 and Δ120–162-Cav-2 mutants but not with Δ1–70-Cav-2 and Δ47–86-Cav-2 mutants (Figure 1C), indicating that the residues 47–70 within the oligomerization domain (residues 47–86) (40) of Cav-2 is the binding motif for lamin A/C. No interaction of Cav-2 with lamin B1 was further confirmed (Figure 1C). These data show that A-type lamin specifically associates with INM-targeted pY19-Cav-2 through direct interaction at the nuclear periphery in response to insulin.

To test if the binding of Cav-2 to lamin A/C affects its mobility within the INM, photoactivation was performed in Δ47–86-Cav-2 mutant-expressed and/or lamin A/C-depleted cells. Photoactivated WT Cav-2 exhibited a slow movement in the NE (mobility rate: 13.1%) (Figure 1D, panels 1), whereas Δ47–86-Cav-2 mutant revealed a rapid movement of the fluorescent signal from the point of photoactivation to the surrounding NE (mobility rate: 51.8%) (Figure 1D, panels 2). Lamin A/C depletion speeded up the movement of Cav-2 (mobility rate: 50.4%) (Figure 1D, panels 3). The movement of Δ47–86-Cav-2 mutant in lamin A/C-depleted cells showed no further significant increase in its mobility (mobility rate: 52.9%) (Figure 1D, panels 4 versus 2), indicating that the lamin A/C interaction is the only factor.

### Requirement of pY19-Cav-2 association with lamin A/C for maintenance of the Tyr-19 phosphorylation in the INM

**Figure 2.**
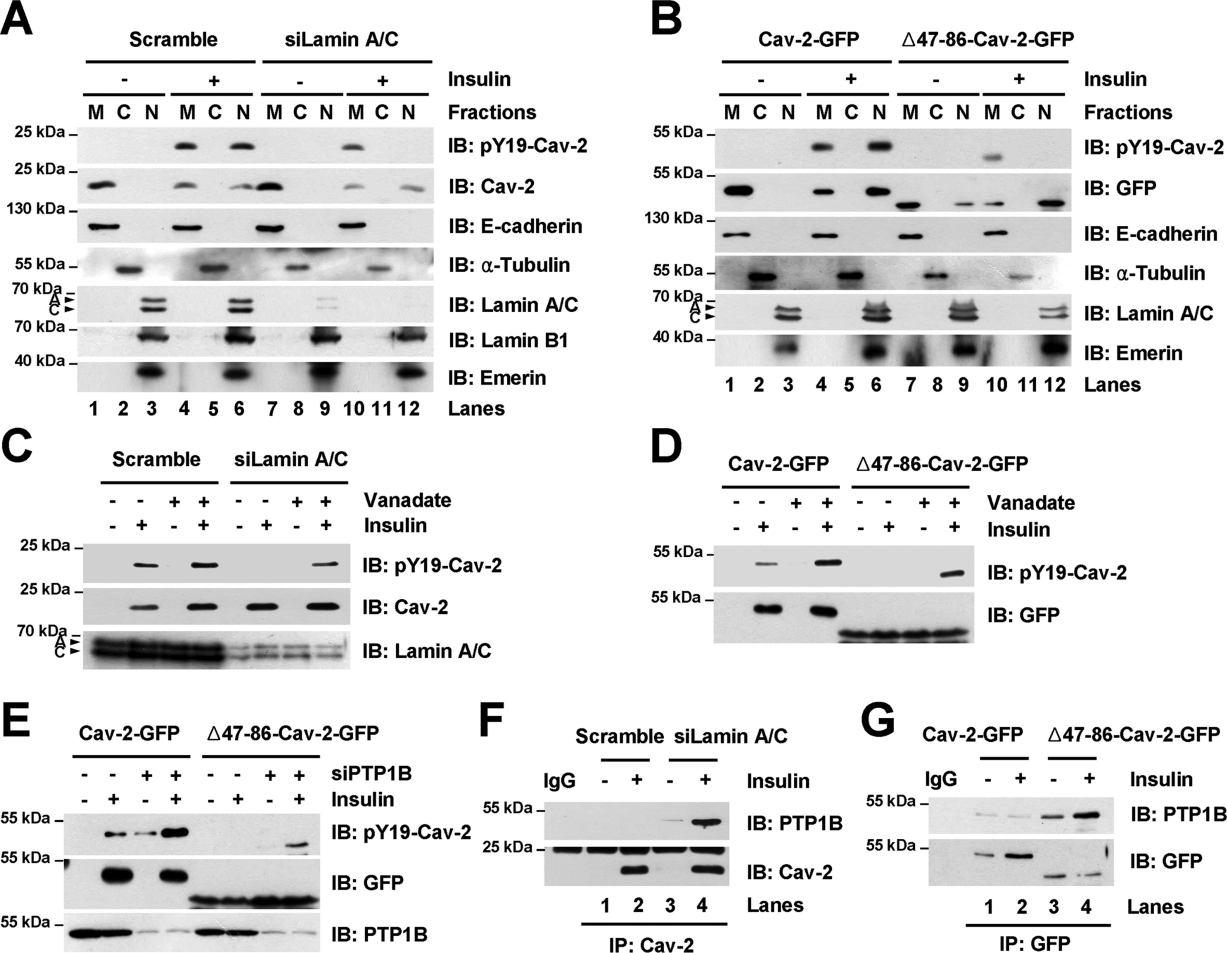
pY19-Cav-2 association with lamin A/C is essential for the sustenance of Tyr-19 phosphorylation from the dephosphorylation by nuclear PTP1B. (**A** and **B**) Lamin A/C association of Cav-2 in the INM for the maintenance of Tyr-19 phosphorylation. Scramble control or siLamin A/C-transfected Hirc-B cells (**A**) or Cav-2-GFP- or Δ47–86-Cav-2-GFP-expressed HEK293T cells (**B**) treated with or without 100 nM insulin for 30 min were subjected to nuclear fractionation. Equal amounts of protein for each fraction were analyzed by immunoblotting (*n* = 3). E-cadherin, α-tubulin, and lamin A/C and emerin were detected as markers for membrane (M), cytoplasmic (C) and nuclear (N) fractions, respectively. (**C** and **D**) Nuclear dephosphorylation of pY19-Cav-2 defective in its association with lamin A/C. Nuclear lysates from scramble control or siLamin A/C-transfected Hirc-B cells (**C**) or Cav-2-GFP- or Δ47–86-Cav-2-GFP-expressed HEK293T cells (**D**) treated with or without 200 μM vanadate for 30 min followed by incubation with or without 100 nM insulin for 30 min were subjected to immunoblotting (*n* = 3). (**E**) Dephosphorylation of pY19-Cav-2 defective in the lamin A/C association by nuclear PTP1B. Nuclear lysates from Cav-2-GFP or Δ47–86-Cav-2-GFP and scramble control or siPTP1B-co-transfected HEK293T cells treated with or without 100 nM insulin for 30 min were subjected to immunoblotting (*n* = 3). (**F** and **G**) Interaction of the lamin A/C association-defective Cav-2 with nuclear PTP1B. Nuclear lysates from scramble control or siLamin A/C-transfected Hirc-B cells (**F**) or Cav-2-GFP- or Δ47–86-Cav-2-GFP-expressed HEK293T cells (**G**) treated with or without 100 nM insulin for 30 min were immunoprecipitated with anti-Cav-2 (**F**) or anti-GFP (**G**) antibody and subjected to immunoblotting (*n* = 3).

We then tested whether Cav-2 association with lamin A/C is important for the Tyr-19 phosphorylation status in the nucleus. Endogenous pY19-Cav-2 was not detected in the nuclear fraction of lamin A/C-depleted cells in response to insulin as compared to scramble control (Figure 2A, lanes 6 versus 12). Tyr-19-phosphorylated Cav-2 was also not detected in the nuclear fraction of Δ47–86-Cav-2 mutant-expressed cells in response to insulin as compared to ectopic WT Cav-2-expressed cells (Figure 2B, lanes 6 versus 12). However, endogenous Cav-2 and ectopic Δ47–86-Cav-2 mutant were localized in their nuclear fractions in response to insulin (Figure 2A and B, lanes 6 versus 12). And insulin-stimulated Tyr-19-phosphorylated endogenous Cav-2 and ectopic Δ47–86-Cav-2 mutant were detected in their membrane fractions (Figure 2A and B, lanes 10), suggesting that the defect in the binding of INM-targeted pY19-Cav-2 to lamin A/C leads to dephosphorylation of the Tyr-19 by a nuclear protein tyrosine phosphatase (PTPase).

To test the dephosphorylation of pY19-Cav-2 by nuclear PTPases, lamin A/C-depleted or Δ47–86-Cav-2 mutant-expressed cells were treated with vanadate, a PTPase inhibitor. Vanadate treatment restored Tyr-19-phosphorylated endogenous Cav-2 and ectopic Δ47–86-Cav-2 mutant in the nuclei in response to insulin (Figure 2C and D). A nuclear PTPase, PTP1B is shown to co-localize with lamin A/C in the INM and dephosphorylate emerin (41). Knockdown of PTP1B by PTP1B siRNA prevented the pY19 dephosphorylation of Δ47–86-Cav-2 mutant (Figure 2E). In agreement with the results, endogenous Cav-2 in lamin A/C-depleted cells and ectopic Δ47–86-Cav-2 mutant, but not the Cav-2 in scramble control and ectopic WT Cav-2 in the nuclei interacted with PTP1B in response to insulin (Figure 2F and G). These results show that the specific interaction of INM-localized pY19-Cav-2 with lamin A/C at the nuclear periphery is important for the maintenance of Tyr-19 phosphorylation of Cav-2 against its dephosphorylation by nuclear PTP1B.

### Regulation of insulin-induced histone H3 modifications at the nuclear periphery by lamin A/C-associated pY19-Cav-2 in the INM

**Figure 3.**
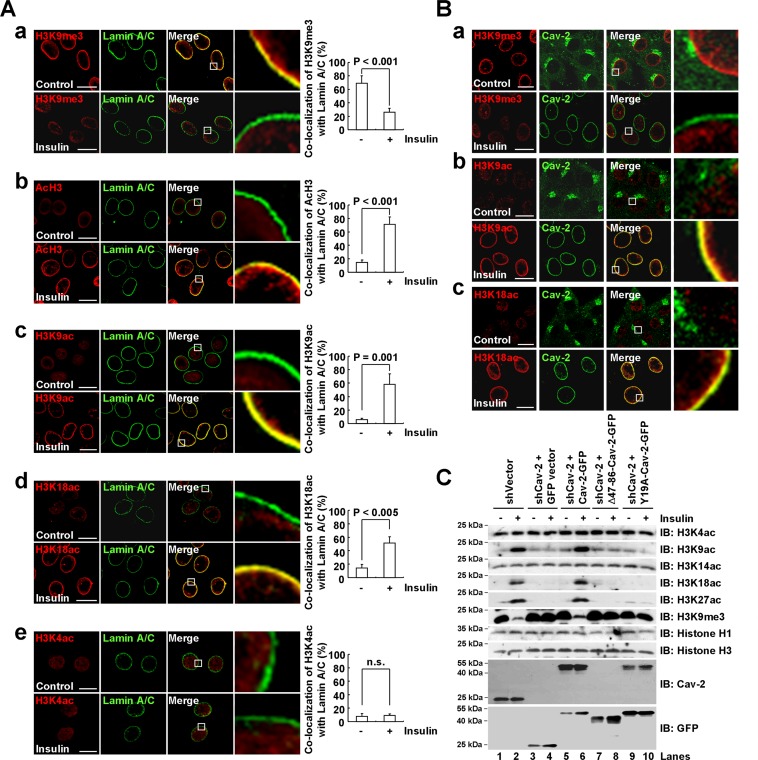
Lamin A/C-associated pY19-Cav-2 in the INM regulates histone H3 modifications at the nuclear periphery in response to insulin. (**A** and **B**) Insulin-induced disassembly of H3K9me3 and assembly of H3K9ac and H3K18ac at the nuclear periphery composed of lamin A/C and Cav-2 in the INM. Hirc-B cells were treated with or without 100 nM insulin for 30 min, stained with anti-H3K9me3 (**A**, **a** and **B**, **a**), anti-AcH3 (**A**, **b**), anti-H3K9ac (**A**, **c** and **B**, **b**), anti-H3K18ac (**A**, **d** and **B**, **c**) or anti-H3K4ac (**A**, **e**) antibody followed by TRITC-conjugated antibody and with anti-lamin A/C (**A**) or anti-Cav-2 (**B**) antibody followed by FITC-conjugated antibody, and analyzed by confocal microscopy (mean ± SE, *n* = 3). The images on the right show magnifications of the areas framed in the Merge images. Scale bars, 20 μm. n.s., nonsignificant. (**C**) Regulation of insulin-induced disassembly of H3K9me3 and assembly of H3K9ac, H3K18ac and H3K27ac by lamin A/C-associated pY19-Cav-2. Whole cell lysates from control shRNA vector-expressed Hirc-B cells or GFP vector-expressed Cav-2 shRNA-, Cav-2-GFP-expressed Cav-2 shRNA-, Δ47–86-Cav-2-GFP-expressed Cav-2 shRNA- or Y19A-Cav-2-GFP-expressed Cav-2 shRNA-stable Hirc-B cells treated with or without 100 nM insulin for 30 min were subjected to immunoblotting (*n* = 4).

While H3K9me3 correlates with gene silencing (25,42), histone H3 lysines 9, 18 and 27 acetylations (H3K9ac, H3K18ac and H3K27ac) are enriched around the TSSs of active genes (43). To verify if lamin A/C-associated Cav-2 localized in the INM regulates insulin-induced histone H3 modifications, confocal imaging analyses were carried out with endogenous Cav-2-expressing Hirc-B cells. Whereas control cells exhibited a nuclear rimming pattern of H3K9me3 heterochromatin staining (Figure 3A, a), insulin stimulation resulted in a loss of the nuclear periphery staining and a concomitant shift toward a more diffuse nuclear-wide pattern in the cells (Figure 3A, a). In contrast, insulin promoted the nuclear periphery stainings of AcH3, H3K9ac and H3K18ac (Figure 3A, b–d) but did not influence the H3K4ac-marked chromatin staining (Figure 3A, e). The nuclear periphery staining of H3K9me3, AcH3, H3K9ac, H3K18ac or H3K4ac-marked chromatin was quantitated by co-merge with lamin A/C. As demonstrated by co-merge of their nuclear periphery stainings with INM-targeted Cav-2, the disassembly of H3K9me3 (Figure 3B, a) and assembly of H3K9ac (Figure 3B, b) and H3K18ac (Figure 3B, c) were detected at the nuclear periphery in response to insulin.

We then tested further the requirement of lamin A/C-associated pY19-Cav-2 for the histone H3 modifications in Cav-2-deficient cells. Insulin induced H3K9ac, H3K18ac and H3K27ac in control cells (Figure 3C, lanes 1 versus 2) but the acetylations were not observed in Cav-2-deficient cells (Figure 3C, lanes 3 versus 4). Reexpression of WT Cav-2 restored the H3K9ac, H3K18ac and H3K27ac (Figure 3C, lanes 4 versus 6), but expression of Δ47–86-Cav-2 or Y19A-Cav-2 mutant exhibited no effect (Figure 3C, lanes 4 versus 8 and 10). Consistently insulin induced the reduction of H3K9me3 in control cells (Figure 3C, lanes 1 versus 2) but the reduction was not observed in Cav-2-deficient cells (Figure 3C, lanes 3 versus 4). And reexpression of WT Cav-2 (Figure 3C, lanes 4 versus 6), but not Δ47–86-Cav-2 or Y19A-Cav-2 mutant (Figure 3C, lanes 4 versus 8 and 10) restored the reduction of H3K9me3. These results show that A-type lamin-associated pY19-Cav-2 in the INM regulates disassembly of H3K9me3 and assembly of H3K9ac, H3K18ac and H3K27ac at the nuclear periphery in response to insulin.

### Dissociation of *Egr-1* and *JunB* promoters from lamin A/C by pY19-Cav-2 in the INM for their insulin-response transactivation at the nuclear periphery

**Figure 4.**
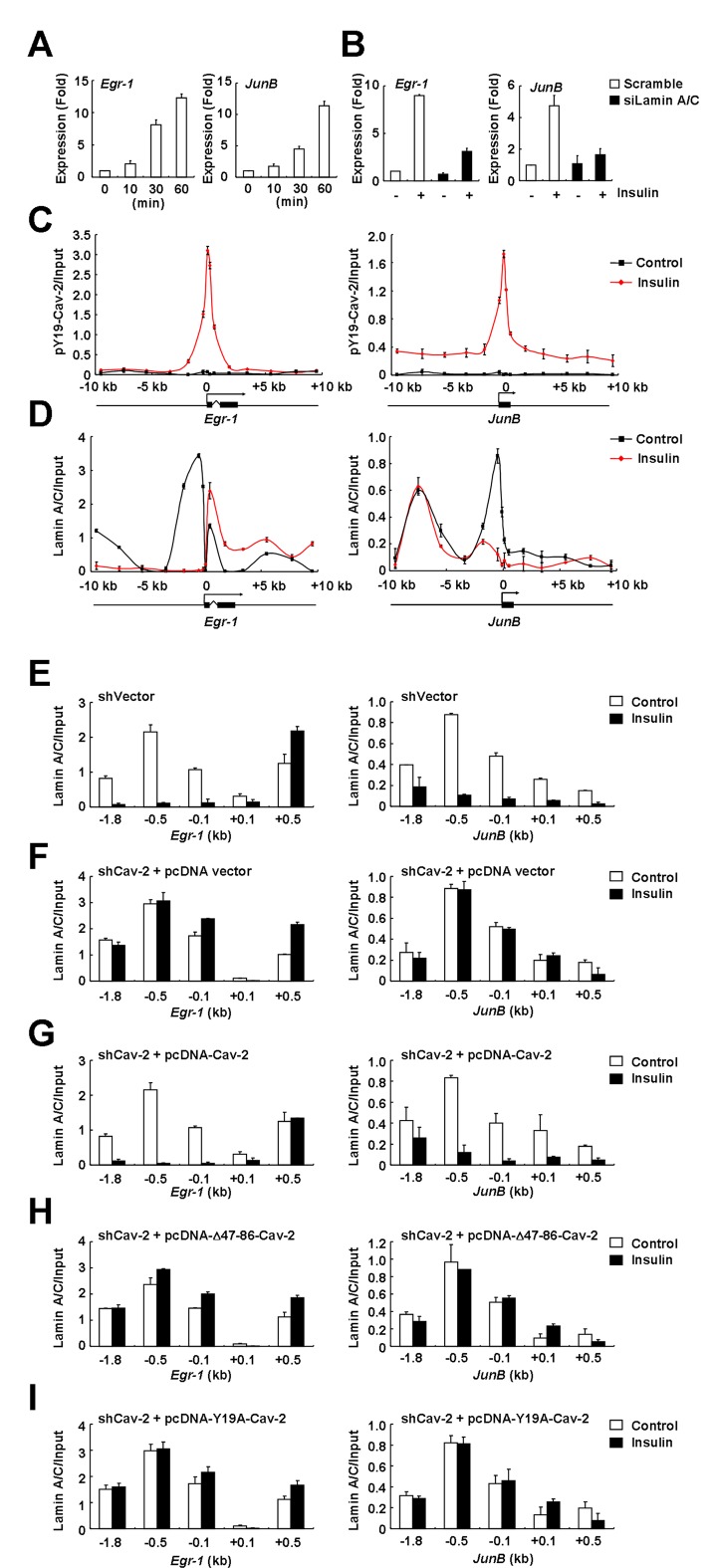
Lamin A/C-associated pY19-Cav-2 promotes dissociation of the *Egr-1* and *JunB* promoters from lamin A/C in response to insulin. (**A**) Insulin-stimulated *Egr-1* and *JunB* induction. Hirc-B cells treated with 100 nM insulin for indicated time points were analyzed for *Egr-1* and *JunB* expression by qRT-PCR (mean ± SE, *n* = 3). (**B**) Inhibition of insulin-induced *Egr-1* and *JunB* expression by lamin A/C knockdown. Scramble or siLamin A/C-transfected Hirc-B cells treated with or without 100 nM insulin for 30 min were analyzed for *Egr-1* and *JunB* expression by qRT-PCR (mean ± SE, *n* = 3). (**C** and **D**) Insulin-induced enrichment of pY19-Cav-2 around the TSSs (−0.5 to +0.5 kb) and disengagement of the promoters (−0.5 to −0.1 kb) from lamin A/C of *Egr-1* and *JunB* genes. Hirc-B cells treated with or without 100 nM insulin for 30 min were subjected to ChIP with anti-pY19-Cav-2 (**C**) or anti-lamin A/C (**D**) antibody on *Egr-1* and *JunB* genes. qPCR data are presented (mean ± SE, *n* = 3). The intron (zigzag line)/exon (black boxes) organization of the genes is shown at the bottom with an arrow indicating the TSS. (**E**–**I**) Insulin-induced disengagement of the inactive *Egr-1* and *JunB* promoters from their lamin A/C binding by A-type lamin-associated pY19-Cav-2. Control shRNA vector-expressed Hirc-B cells (**E**) or pcDNA vector-expressed Cav-2 shRNA- (**F**), pcDNA-Cav-2-expressed Cav-2 shRNA- (**G**), pcDNA-Δ47–86-Cav-2-expressed Cav-2 shRNA- (**H**) or pcDNA-Y19A-Cav-2-expressed Cav-2 shRNA-stable (**I**) Hirc-B cells treated with or without 100 nM insulin for 30 min were subjected to ChIP with anti-lamin A/C antibody on *Egr-1* and *JunB* genes. qPCR data are presented (mean ± SE, *n* = 3).

pY19-Cav-2 transactivated STAT3-mediated insulin-response *Egr-1* and *JunB* induction (8). qRT-PCR analysis consistently showed insulin-stimulated *Egr-1* and *JunB* expression (Figure 4A). Lamin A/C depletion, resulted in the Tyr-19 dephosphorylation of Cav-2 in the INM (Figure 2), caused significant decreases in insulin-induced *Egr-1* and *JunB* expression (Figure 4B). When ChIP analysis of pY19-Cav-2 on the *Egr-1* and *JunB* genes was performed to verify insulin-induced pY19-Cav-2 association with and recruitment of the promoters in the INM, a dramatic enrichment of pY19-Cav-2 around TSSs (−0.5 to +0.5 kb) of the genes was observed in response to insulin (Figure 4C). The robust enrichment of pY19-Cav-2 around the TSSs was in parallel with the high level of RNA Pol II recruitment in the regions (Supplementary Figure S2A), suggesting that these insulin-induced changes were linked. ChIP with anti-Cav-2 antibody showed a large transient increase around the TSSs (Supplementary Figure S2B) with its kinetics similar to that of pY19-Cav-2 and verified that the Cav-2 enriched around the TSSs is Tyr-19-phosphorylated. Adipogenic differentiation resulted in disengagement of adipogenic loci from A-type lamins and their transcriptional activation (39). Thus, we examined if the enrichment of pY19-Cav-2 around the TSSs and INM-targeted pY19-Cav-2 interaction with lamin A/C change the lamin association of *Egr-1* and *JunB* genes in response to insulin. ChIP analyses of lamin A/C on the *Egr-1* and *JunB* genes showed that the promoter regions (−0.5 to −0.1 kb) of repressed *Egr-1* and *JunB* genes were associated with lamin A/C in the absence of insulin but insulin treatment led to disengagement of the promoters from lamin A/C (Figure 4D and E). The dissociation did not occur in Cav-2-deficient cells as compared to vector control cells (Figure 4E versus F). However, Cav-2-deficient cells reexpressed with ectopic WT Cav-2 exhibited the dissociation of *Egr-1* and *JunB* promoters from lamin A/C in response to insulin (Figure 4G), but Δ47–86-Cav-2 and Y19A-Cav-2 mutants had no effect (Figure 4H and I). These results show that A-type lamin-associated pY19-Cav-2 in the INM facilitates disengagement of the inactive promoters of *Egr-1* and *JunB* from their lamin A/C association.

**Figure 5.**
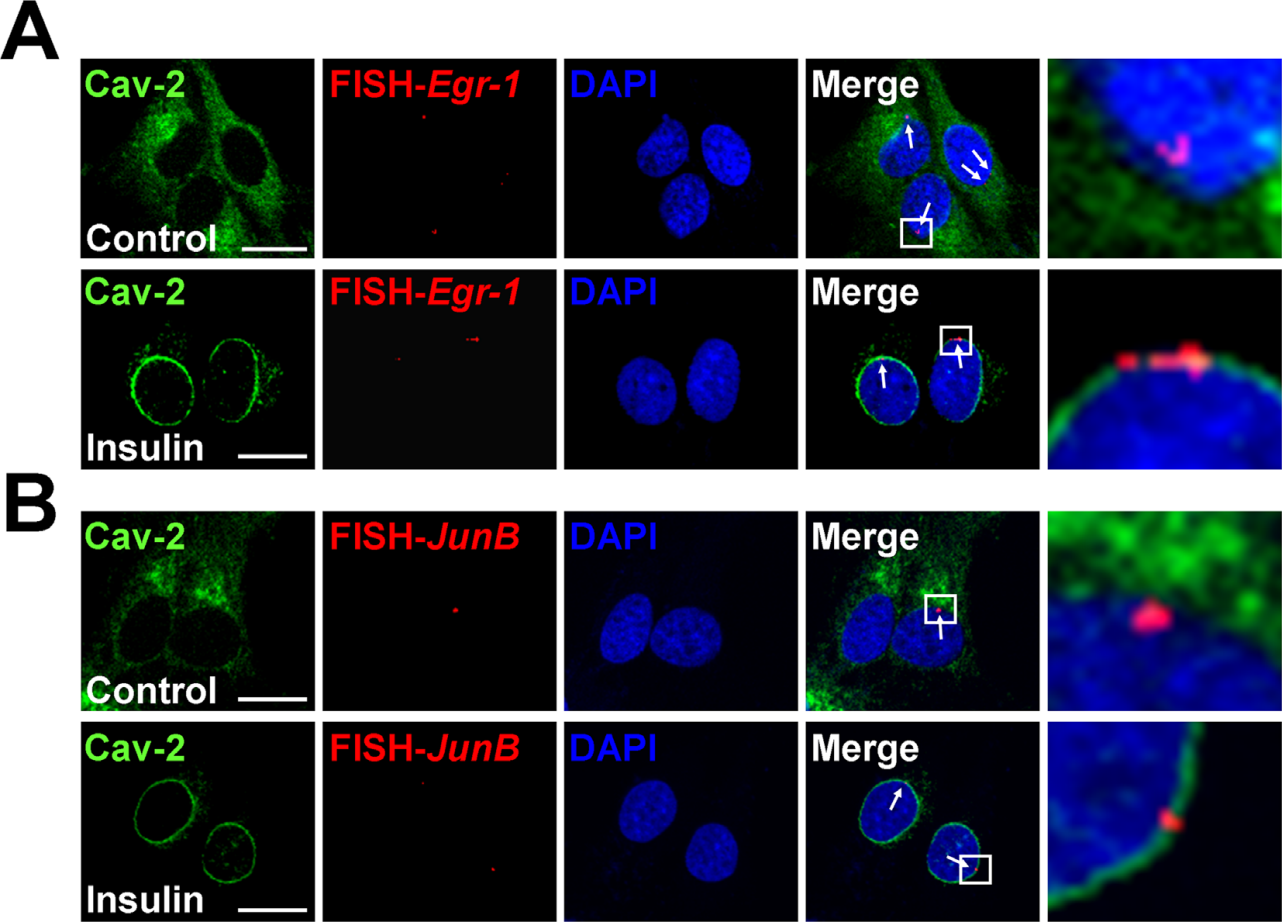
*Egr-1* and *JunB* genes are positioned at nuclear periphery and co-localize with the INM-targeted Cav-2 in response to insulin. The *in situ* localization of *Egr-1* and *JunB* genes at the nuclear periphery was determined by FISH in Hirc-B cells treated with or without 100 nM insulin for 30 min. Representative images of single confocal sections of nuclei show the immunostained Cav-2 (green), FISH signal (red) and DAPI (blue). The arrows in the merge panel indicate the positive signals in the Alexa Fluor 594-labeled *Egr-1* (**A**) and *JunB* (**B**) specific DNA probes. The images on the right show magnifications of the areas framed in the merge images. Scale bars, 20 μm.

To determine *in situ* nuclear localization of *Egr-1* and *JunB* genes and to test whether the pY19-Cav-2-mediated dissociation of the promoters from lamin A/C (Figure 4) causes relocation of the genes to the nuclear interior, FISH analysis was performed. *Egr-1* and *JunB* genes were located at the nuclear periphery in the basal condition (Figure 5A and B, top panels). Upon insulin stimulation, no nuclear interior localization of the genes was detected and the position of *Egr-1* and *JunB* genes at the nuclear periphery overlapped with the fluorescence signal of INM-targeted Cav-2 from Golgi (6) (Figure 5A and B, bottom panels). These results show that the *Egr-1* and *JunB* genes localize with Cav-2 at the INM after insulin treatment.

### Insulin-induced enrichment of H3K9ac and H3K18ac via recruitment of GCN5 and p300 on the *Egr-1* and *JunB* promoters at the nuclear periphery by lamin A/C-associated pY19-Cav-2 in the INM

**Figure 6.**
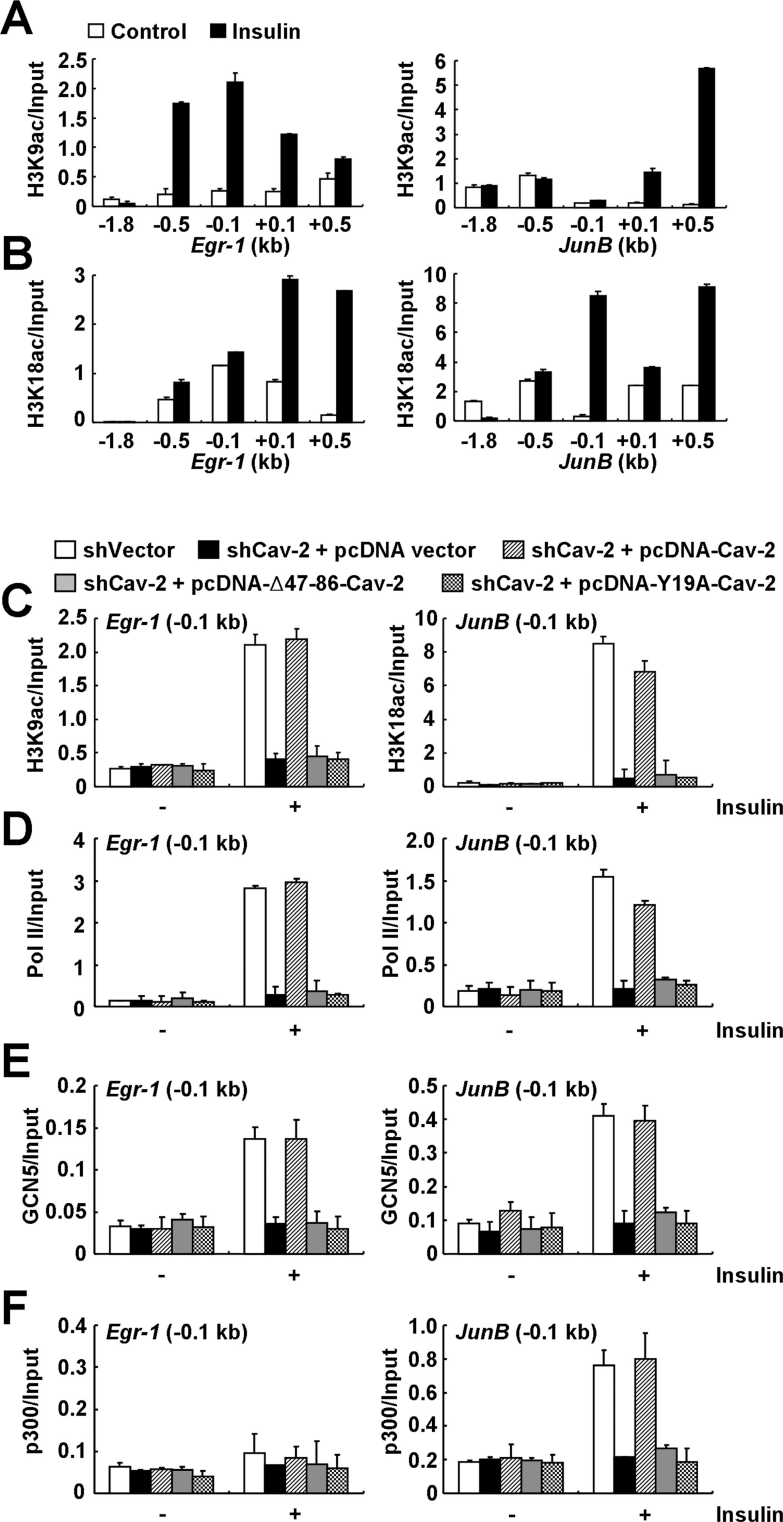
Lamin A/C-associated pY19-Cav-2-mediated recruitment of GCN5 and p300 precedes the subsequent enrichment of H3K9ac and H3K18ac and RNA Pol II on the *Egr-1* and *JunB* promoters in response to insulin. (**A** and **B**) Insulin-induced enrichment of H3K9ac and H3K18ac on the *Egr-1* and *JunB* promoters (−0.5 to −0.1 kb). Hirc-B cells treated with or without 100 nM insulin for 30 min were subjected to ChIP with anti-H3K9ac (**A**) or anti-H3K18ac (**B**) antibody on *Egr-1* and *JunB* genes. qPCR data in all figures are presented (mean ± SE, *n* = 4). (**C** and **D**) Regulation of the enrichment of H3K9ac on *Egr-1* and H3K18ac on *JunB* promoters and the RNA Pol II recruitment to the promoters (-0.1 kb) by lamin A/C-associated pY19-Cav-2. Control shRNA vector-expressed Hirc-B cells or pcDNA vector-expressed Cav-2 shRNA-, pcDNA-Cav-2-expressed Cav-2 shRNA-, pcDNA-Δ47–86-Cav-2-expressed Cav-2 shRNA- or pcDNA-Y19A-Cav-2-expressed Cav-2 shRNA-stable Hirc-B cells treated with or without 100 nM insulin for 30 min were subjected to ChIP with anti-H3K9ac or anti-H3K18ac antibody (**C**) or anti-RNA Pol II (**D**) antibody on the promoters of *Egr-1* and *JunB* genes. qPCR data are presented (mean ± SE, *n* = 4). (**E** and **F**) Recruitment of GCN5 and p300 on the *Egr-1* and *JunB* promoters by lamin A/C-associated pY19-Cav-2. Control shRNA vector-expressed Hirc-B cells or pcDNA vector-expressed Cav-2 shRNA-, pcDNA-Cav-2-expressed Cav-2 shRNA-, pcDNA-Δ47–86-Cav-2-expressed Cav-2 shRNA- or pcDNA-Y19A-Cav-2-expressed Cav-2 shRNA-stable Hirc-B cells treated with or without 100 nM insulin for 30 min were subjected to ChIP with anti-GCN5 (**E**) or anti-p300 (**F**) antibody on the promoters of *Egr-1* and *JunB* genes. qPCR data are presented (mean ± SE, *n* = 4).

We further investigated if lamin A/C-associated pY19-Cav-2 in the INM participates in the histone H3 modifications for insulin-response epigenetic activation of *Egr-1* and *JunB* at the nuclear periphery by ChIP analysis. Insulin stimulation decreased H3K9me3 and increased AcH3 around the TSSs of the genes (Supplementary Figure S2C and D), and specifically increased H3K9ac on *Egr-1* and H3K18ac on *JunB* promoter regions (Figure 6A and B). H3K27ac was increased around the TSS of *JunB* in response to insulin (Supplementary Figure S3A). Insulin treatment had little effect on the levels of H3K14ac and H3K4ac within the regions of both genes (Supplementary Figure S3B and C). Total histone H3 signals on *Egr-1* and *JunB* genes were not influenced by insulin treatment (Supplementary Figure S3D). ChIP with anti-IgG antibody showed no signal (Supplementary Figure S3E). No enrichment of Cav-2 or pY19-Cav-2 around the TSSs of a housekeeping gene, *Actb* was detected in response to insulin (Supplementary Figure S3F). Insulin-induced enrichment of H3K9ac on *Egr-1* and H3K18ac on *JunB* promoters was abrogated in Cav-2-deficient cells (Figure 6C). Reexpression of WT Cav-2 restored the H3K9ac and H3K18ac enrichment but Δ47–86-Cav-2 or Y19A-Cav-2 mutant had no effect (Figure 6C). In parallel, Cav-2 depletion prevented insulin-induced enrichment of AcH3 and RNA Pol II on the promoters and the inhibition was rescued by ectopic WT Cav-2 but not Δ47–86-Cav-2 or Y19A-Cav-2 mutant (Figure 6D and Supplementary Figure S4A).

GCN5 and p300 play important roles in the regulation of gene expression by modifying chromatin and serving as transcription coactivators (44,45). To test whether GCN5 and p300 are responsible for the lamin A/C-associated pY19-Cav-2-mediated histone H3 acetylations, effects of butyrolactone 3 and curcumin, inhibitors of GCN5 and p300, respectively (46,47) were examined. Enrichment of H3K9ac on *Egr-1* and H3K18ac on *JunB* promoters in response to insulin was inhibited by butyrolactone 3 treatment, whereas the enrichment of H3K18ac on *JunB* but not H3K9ac on *Egr-1* promoter was attenuated by curcumin treatment (Supplementary Figure S4B). Inhibition of insulin-induced RNA Pol II recruitment to the promoters coincided with the abrogation in the enrichment of H3K9ac on *Egr-1* and H3K18ac on *JunB* promoters by the inhibitor treatments (Supplementary Figure S4C). Finally, we tested the requirement of lamin A/C-associated pY19-Cav-2 for the recruitment of GCN5 and p300 to the *Egr-1* and *JunB* promoters and the subsequent acetylations of H3K9 and H3K18 in response to insulin in Cav-2-deficient cells. Whereas insulin induced recruitment of GCN5 to the *Egr-1* and *JunB* promoters (Figure 6E) and of p300 to the *JunB* but not *Egr-1* promoter (Figure 6F) in control cells, the recruitment was abrogated in Cav-2-deficient cells. Reexpression of ectopic WT Cav-2 restored insulin-induced recruitment of GCN5 and p300 on the promoters but Δ47–86-Cav-2 or Y19A-Cav-2 mutant had no effect (Figure 6E and F). Together these data show that the recruitment of GCN5 and p300 by A-type lamin-associated pY19-Cav-2 in the INM precedes the subsequent sequential enrichment of H3K9ac and H3K18ac by GCN5 and/or p300 and RNA Pol II on the *Egr-1* and *JunB* promoters to initiate insulin-response epigenetic transcription of the target genes at the nuclear periphery.

## DISCUSSION

The functional role of INM proteins is not well-defined and the regulatory mechanisms by INM proteins in histone modifications have been unclear. INM proteins including LBR, lamina-associated polypeptide (LAP) 1, LAP2, emerin, MAN1 and nurim are important components of nuclear lamina. Most of the INM proteins interact with the lamins and heterochromatin (48). LBR, a B-type lamin-associated INM protein (49,50), is reported to induce chromatin compaction and transcriptional repression by binding to methylated H3K9, H3K27 or H4K20 (51–53). Emerin has been shown to interact with lamin A/C (54,55) and facilitate repressive chromatin formation at the nuclear periphery by increasing the catalytic activity of histone deacetylase 3 (HDAC3) (56). LAP2β binds to HDAC3 (57) and influences the levels of histone H4 acetylation (58). Collectively these reports suggest that INM proteins modulate epigenetic modification of chromatin through interaction with certain epigenetic factors and/or histone modifying enzymes which are involved in the transcriptional inactivation.

Thus far, the regulatory mechanisms by which a specific INM protein activates gene transcription at the nuclear periphery via its association with promoter, chromatin, epigenetic modifying enzymes, RNA Pol II, and nuclear lamins have not been identified. Our findings show that Cav-2, a novel INM protein, associated specifically with the A-type lamin activates insulin-response genes at the nuclear periphery where Tyr-19-phosphorylated Cav-2 disengages the inactivated *Egr-1* and *JunB* promoters from the lamin A/C through the disassembly of H3K9me3 and facilitates the assembly of H3K9ac, H3K18ac and H3K27ac by the recruitment of GCN5 and p300 and the subsequent enrichment of RNA Pol II on the target gene promoters. Thus, the present study provides new mechanistic insights into the spatiotemporal dynamics of organization and arrangement of chromatin and chromatin histone modifications for epigenetic transcriptional activation at the nuclear periphery by INM-integrated proteins in association with specific nuclear lamin. It will be of interest to identify other INM proteins which interact with specific A- or B-type lamin and involve in the regulation of gene activation at the nuclear periphery.

Cav-2 exists in an oligomeric form in the PM microdomain (33). Our data show that A-type lamin binds to residues 47–70 within the oligomerization domain (residues 47–86) (40) of Cav-2 in the INM, and that INM-targeted Δ47–86-Cav-2 mutant does not interact with lamin A/C and is dephosphorylated by nuclear PTP1B and has no regulatory function in the epigenetic activation of insulin-response *Egr-1* and *JunB* genes. Thus our findings suggest that oligomeric status of Cav-2 in the INM seems to be important for the epigenetic regulation by the lamin A/C-associated pY19-Cav-2 at the nuclear periphery. Further work is necessary to identify the possible hierarchical or reciprocal relationship between oligomerization and phosphorylation and association with lamin A/C of Cav-2 in the INM. Nevertheless, the present study delineates novel molecular mechanisms of insulin-response epigenetic activation at the nuclear periphery, which are controlled by the phosphorylation and association with the A-type lamin and possibly oligomerization of Cav-2 in the INM.

Insulin is one of the adipogenic hormones required for 3T3L1 preadipocytes and adipose stem cells (ASCs) to differentiate into adipocytes (39,59). *Egr-1* and *JunB* were identified as insulin-response immediate early genes induced by adipogenic stimuli via mitogen-activated protein kinase and protein kinase C pathways in 3T3L1 preadipocytes (59). However, epigenetic regulation of the genes by epigenetic factors in specific nuclear territory has not been elucidated. Recently, Lund *et al*. (39) showed that in an adipogenesis system using ASCs, adipogenic differentiation results in dissociation of adipogenic loci from the lamin A/C and their transactivation. Our present study shows that INM-targeted pY19-Cav-2 facilitates disengagement of *Egr-1* and *JunB* genes from lamin A/C for epigenetic transcriptional activation in response to insulin. Thus, our findings not only provide a novel regulatory mechanism for epigenetic modulation of insulin-response genes by Cav-2 at the nuclear periphery, but also suggest that Cav-2 might control the interaction between insulin-response adipogenic genes and lamin A/C for the activation of the genes in adipogenesis.

## SUPPLEMENTARY DATA

Supplementary Data are available at NAR Online.

SUPPLEMENTARY DATA
