# Supplementary material for: Insulin-response epigenetic activation of Egr-1 and JunB genes at the nuclear periphery by A-type lamin-associated pY19-Caveolin-2 in the inner nuclear membrane
Source: Nucleic Acids Res. 2015 Mar 9;43(6):3114–27. doi: 10.1093/nar/gkv181 (PMC4381080; doi:10.1093/nar/gkv181)
Supplement: SUPPLEMENTARY DATA [file supp_43_6_3114__index.html]

Insulin-response epigenetic activation of Egr-1 and JunB genes at the nuclear periphery by A-type lamin-associated pY19-Caveolin-2 in the inner nuclear membrane — Insulin-response epigenetic activation of Egr-1 and JunB genes at the nuclear periphery by A-type lamin-associated pY19-Caveolin-2 in the inner nuclear membrane — SUPPLEMENTARY DATA 

# Insulin-response epigenetic activation of *Egr-1* and *JunB* genes at the nuclear periphery by A-type lamin-associated pY19-Caveolin-2 in the inner nuclear membrane

## SUPPLEMENTARY DATA

**Files in this Data Supplement:**

- SUPPLEMENTARY DATA
