# Supplementary material for: Insulin-response epigenetic activation of Egr-1 and JunB genes at the nuclear periphery by A-type lamin-associated pY19-Caveolin-2 in the inner nuclear membrane
Source: Nucleic Acids Res. 2015 Mar 9;43(6):3114–27. doi: 10.1093/nar/gkv181 (PMC4381080; doi:10.1093/nar/gkv181)

Insulin-response epigenetic activation of *Egr-1* and *JunB* genes at the nuclear periphery by A-type lamin-associated pY19-Caveolin-2 in the inner nuclear membrane

Kyuho Jeong, Hayeong Kwon, Jaewoong Lee, Donghwan Jang and Yunbae Pak

Supplementary Information

Supplementary Table 1

Supplementary Figure Legends

Supplementary Figures S1-4

Supplementary Table S1: List of primers for qPCR

qRT-PCR primers for gene expression analysis:

Gene	Forward primer	Reverse primer
Egr-1	CTGCTTCATCGTCTTCCTCTG	GTCAGTGTTGGGAGTAGGAAAG
JunB	ACACCAACCTCAGCAGTTAC	AAGGGTGGTGCATGTGG
GAPDH	GTCAAGGCTGAGAATGGGAA	ATACTCAGCACCAGCATCAC

qPCR primers for ChIP:

Genomic location	Forward primer	Reverse primer
Egr-1 -9.5 kb	TCAGTCAGCAGAGCGTTTC	CACAGGAAGAGAGCCTTGATAG
Egr-1 -7.5 kb	CCTGTACCCAGTGCCAAAT	CCTACACACACACACCTACAC
Egr-1 -5.5 kb	TGAGAGGGATAGACAGAAAGAGG	AGTATGCTGGTTCACATGGTTAG
Egr-1 -3.4 kb	GCAACGGAGTAGGATACAGATTAG	TTGCTGGGAACCAAACCA
Egr-1 -1.8 kb	GCTCTGTGAAGGAAGTGTTACC	TCTAAGGCTCTCCTGGCC
Egr-1 -0.5 kb	TCTTGGATGGGAGGTCTTCA	TGGGATCTCTCGCGACTC
Egr-1 -0.1 kb	TGTAACCCGGCCAACATC	CTGCAGCGGAGACATCAA
Egr-1 +0.1 kb	CAGCGGCGGCAATAACA	TGTGGTCAGGTGCTCGTA
Egr-1 +0.5 kb	AACTCAAGTTGCGTGGGT	TGACAGATTTCAGCCCTTCC
Egr-1 +1.8 kb	AGGAGTGATGAACGCAAGAG	GGGTAGGAAGAGAGGGAAGA
Egr-1 +3.4 kb	CCATGGGATATGTGGTGTGTATC	TTAGGCAGGAACGGCTACT
Egr-1 +5.5 kb	AGAAACAAGTAAAGGGAGAGAAACA	AGCCTGGACAGCTAGAGAAA
Egr-1 +7.7 kb	GGGCCTTGCGCTTCCTA	GGAGTTGGTGGAAAGAGGACAT
Egr-1 +9.5 kb	CACAAGGTGCTGCGTTTAAT	TACAGTGGTTGCCTGAGAAG

JunB -9.5 kb	GTTGCCATTGTGACTCCTCT	GACTTGGATACTCAGTGTGTGG
JunB -7.3 kb	CGTGCGTGCGAGTACAA	TTTCACCTGCGCTCCAAA
JunB -5.3 kb	GTGTCACAGTCACCTACATACC	GTGAGACCCTGCCTTCAAA
JunB -3.4 kb	GATCTGTAGCTCAGGCAGTATG	CCTACCATTCTCACTCTTGTGG
JunB -1.8 kb	GGACCCTAGACCTTCTGGTAG	GGGCGGGATAATGCTTTCA
JunB -0.5 kb	CAGACTCATCTGTGGGCTTTAG	GCTTAAAGAACAGGTGGGAGAG
JunB -0.1 kb	CTTCCGCAGCTGACAAATTC	CTTTCTCAGCGTCTCTTCTCTC
JunB +0.1 kb	CCCGGATGTGCACGAAA	GGAGTTTGTAGTCGTGAAGAGAA
JunB +0.5 kb	CTCAACCTGGCAGATCCTTATC	TGGAGGCTAGCTTCAGAGAT
JunB +1.8 kb	TGGTCTCTTTCTTCCAAGCTG	CTGGACCTTAAGATACCCGAAAG
JunB +3.4 kb	GGAAAGGAATGGTCAGCCATAG	GCCTCACACCTATCTCTCTCTT
JunB +5.5 kb	CTGGAACTACAGGCATGTATCA	TAGGAGATGAAGCAGGGAGA
JunB +7.3 kb	CAGGACCTAAGAACCTGTCTTT	GCTCAGTGGTTAAGGGTACTT
JunB +9.5 kb	ATCCAAGAGAGGTGGGAGAA	CTGACTCCATAATTCGGGTAGC
Actb -9.5 kb	GCGTGTGTGTATGAGTGTGT	CAGAGTTCAGTTCCCAGCATC
Actb -7.5 kb	TCCTAGGCAAGCGCTCTA	AGCTGGCCTATCTAGTGAGT
Actb -5.5 kb	AGTGGAGCATGCTTCTAATCG	TGAGACAGCTCGTGTTTGAG
Actb -3.4 kb	TCACTCTGTCAGTCCCATCT	CACACAGCAGGATGTCAGTAA
Actb -1.8 kb	GGTCCTGCCTGGGAATTTAG	GGAGAAGATGCCATCCATCAA
Actb -0.5 kb	AGATCTGGCTTTCCTGACTATTG	GACCCTATTTGAGTGGCCTTT
Actb -0.1 kb	ACTCTTCATCCTCTTCCTCAATC	CGCACAGTGCAGCATTT
Actb +0.1 kb	AACTTTACCTTGGCCACTACC	CACCCTAGGCGGAAAGTTAAG
Actb +0.5 kb	CGCTCTATCACTGGGCATT	CGCGCTCCTAGTCTCAATAC

Actb +1.8 kb	TGATACTCCCAGCACACTTAAC	AGCTCCTAAAGCCACAAGAAA
Actb +3.4 kb	TCTGAATGGTCAGCCATTGT	CCTCAACACCTCAAACCACT
Actb +5.5 kb	GCAGTCTACCTTCAACCCTTT	CTACAGCTATTGTCGTCGTTGT
Actb +7.4 kb	CGTTGCAGTAAACACATTAGCC	GTGCAGACTCAGGAACTCATATAC
Actb +9.5 kb	TGTGTCTCCAGGTTTGATGG	GACTGACCTGAAGCTTACTTACT

Supplementary Figure Legends

Figure S1. Solubilization of nuclear lamins.

The purified nuclei from Hirc-B cells were incubated with lysis buffer A (2% Triton X-100, 20 mM Tris-HCl, pH 7.5, 280 mM NaCl, 10 mM NaF, 1mM sodium ortho-vanadate, 5 μg/ml aprotinin, 3 μg/ml pepstatin, 5 μg/ml leupeptin, 1 mM EDTA, and 1 mM DTT) or lysis buffer A containing 6M or 8M urea or 1% SDS for 30 min at room temperature. The lysates were centrifuged at 12,000 rpm for 10 min at 4 °C and the supernatant and pellet were resuspended in 2× SDS-PAGE sample buffer. Equal volume of the resuspended supernatant (S) and pellet (P) were subjected to immunoblotting (*n*=3).

Figure S2. Insulin induces disassembly of H3K9me3 and enrichment of Cav-2, AcH3, and RNA Pol II around the TSSs of *Egr-1* and *JunB* genes.

Hirc-B cells treated with or without 100 nM insulin for 30 min were subjected to ChIP with anti-RNA Pol II (**A**), anti-Cav-2 (**B**), anti-H3K9me3 (**C**) or anti-AcH3 (**D**) antibody on *Egr-1* and *JunB* genes. qPCR data in all figures are presented (mean±SE, *n*=4). The intron (zigzag line)/exon (black boxes) organization of the genes is shown at the bottom with an arrow indicating the TSS.

Figure S3. Insulin treatment increases H3K27ac around the TSSs of *JunB* gene with no effect on H3K14ac, H3K4ac, and total H3 signals on the *Egr-1* and *JunB* genes.

(**A**-**E**) Hirc-B cells treated with or without 100 nM insulin for 30 min were subjected to

ChIP with anti-H3K27ac (**A**), anti-H3K14ac (**B**), anti-H3K4ac (**C**), anti-histone H3 (**D**) or anti-IgG (**E**) antibody on *Egr-1* and *JunB* genes. qPCR data in all figures are presented (mean±SE, *n*=4).

(**F**) No signals of Cav-2 and pY19-Cav-2 around the TSSs of a housekeeping gene, *Actb*. Hirc-B cells treated with or without 100 nM insulin for 30 min were subjected to ChIP with anti-Cav-2 or anti-pY19-Cav-2 antibody on *Actb* gene. qPCR data in all figures are presented (mean±SE, *n*=4). The intron (zigzag line)/exon (black boxes) organization of the genes is shown at the bottom with an arrow indicating the TSS.

Figure S4. Lamin A/C-associated pY19-Cav-2 promotes enrichment of AcH3 on the *Egr-1* and *JunB* promoters, and GCN5 and p300 regulate enrichment of H3K9ac and H3K18ac and RNA Pol II on the promoters in response to insulin.

(**A**) Regulation of the enrichment of AcH3 on *Egr-1* and *JunB* promoters by lamin A/C-associated pY19-Cav-2. Control shRNA vector-expressed Hirc-B cells or pcDNA vector-expressed Cav-2 shRNA-, pcDNA-Cav-2-expressed Cav-2 shRNA-, pcDNA-Δ47-86-Cav-2-expressed Cav-2 shRNA-, or pcDNA-Y19A-Cav-2-expressed Cav-2 shRNA-stable Hirc-B cells treated with or without 100 nM insulin for 30 min were subjected to ChIP with anti-AcH3 antibody on the promoters of *Egr-1* and *JunB* genes. qPCR data are presented (mean±SE, *n*=4).

(**B**,**C**) Acetylation of H3K9 on the *Egr-1* and H3K18 on the *JunB* promoters by GCN5 and H3K18 on the *JunB* promoter by p300, and enrichment of RNA Pol II by GCN5 and p300. Hirc-B cells pretreated with or without 300 µM butyrolactone 3 for 16 h or 50 µM curcumin for 6 h followed by incubation with or without 100 nM insulin for 30 min were subjected to ChIP with anti-H3K9ac or anti-H3K18ac

antibody (**B**), or anti-RNA Pol II antibody (**C**) on the promoters of *Egr-1* and *JunB* genes. qPCR data are presented (mean±SE, *n*=4).

Jeong et al. Figure S1

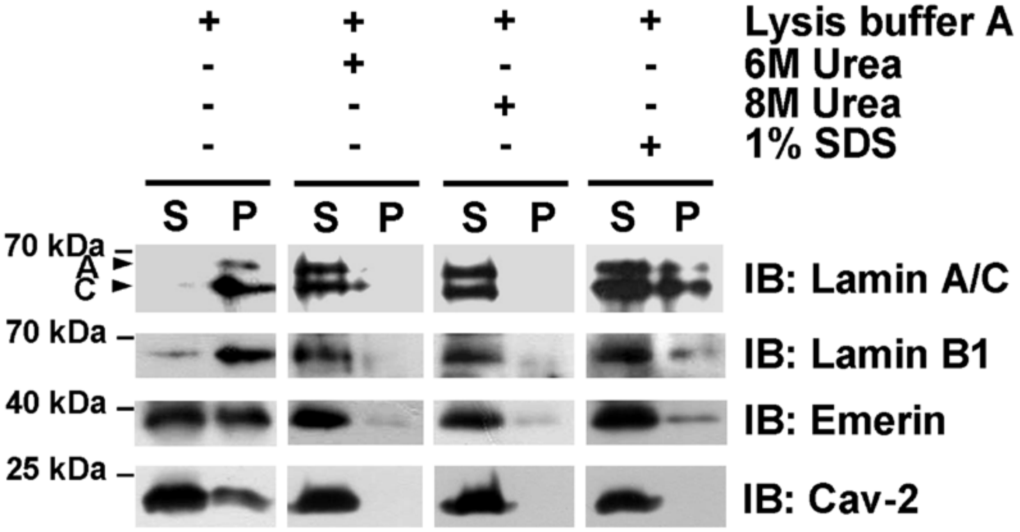

Jeong et al. Figure S2

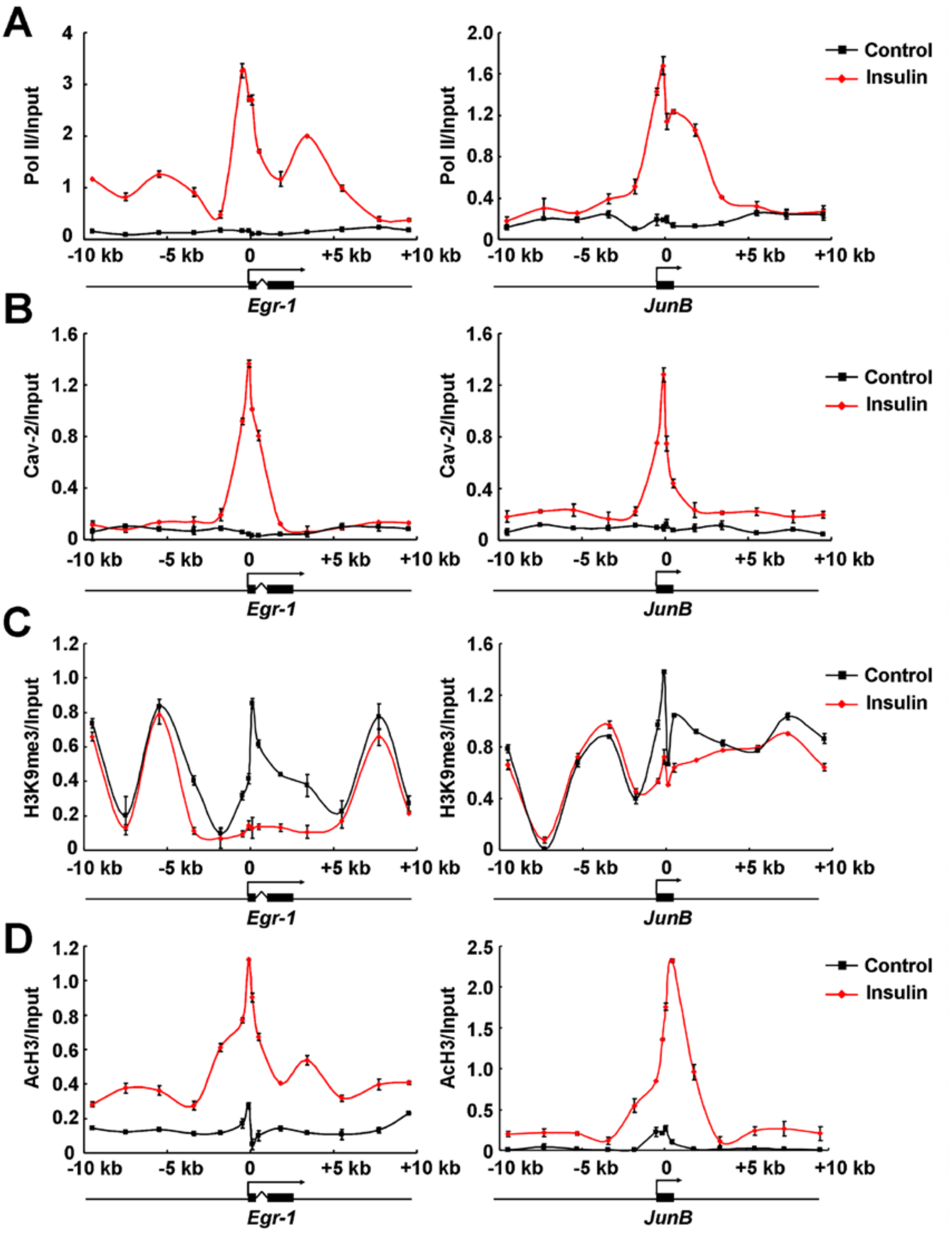

Jeong et al. Figure S3

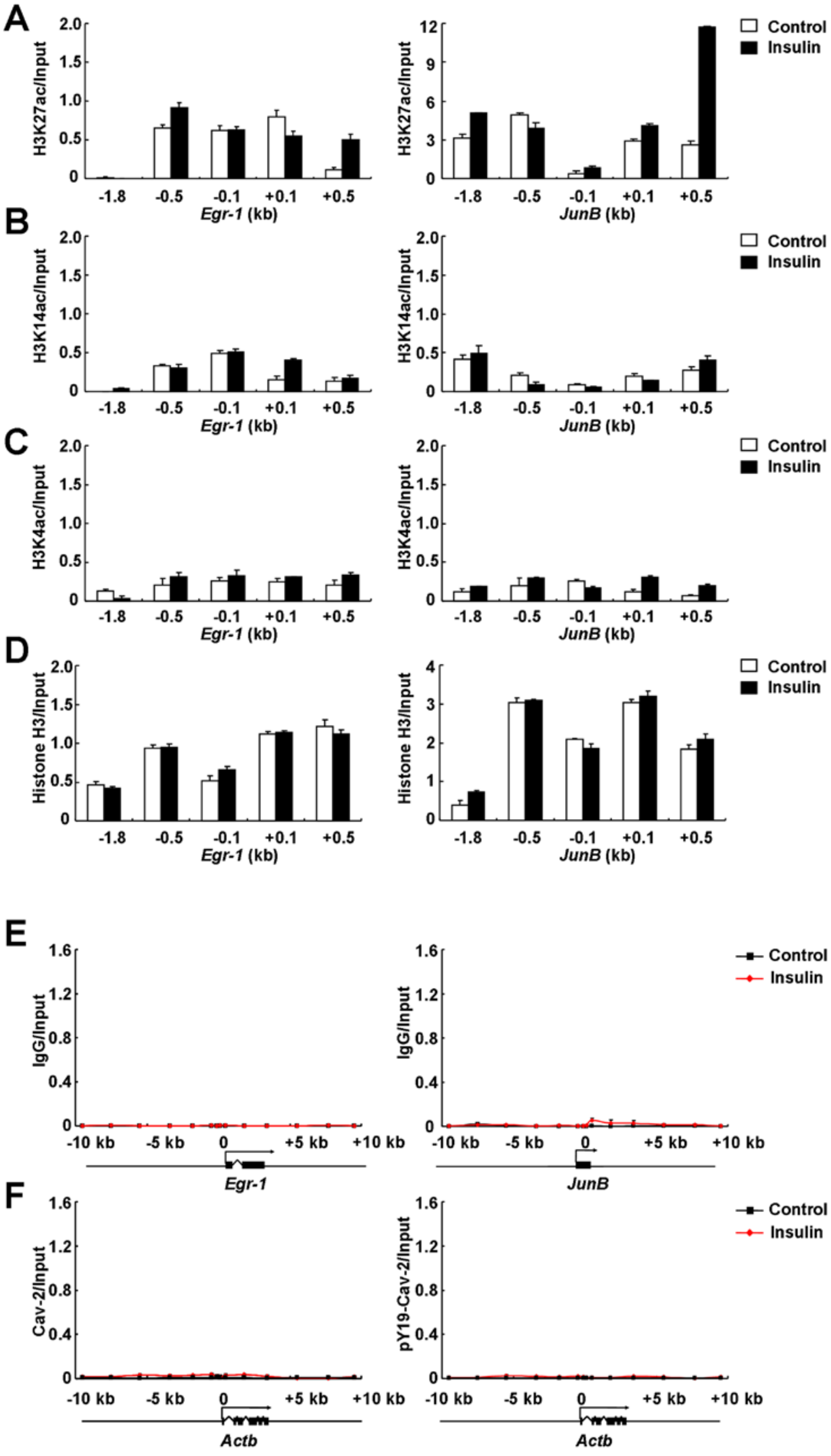

Jeong et al. Figure S4

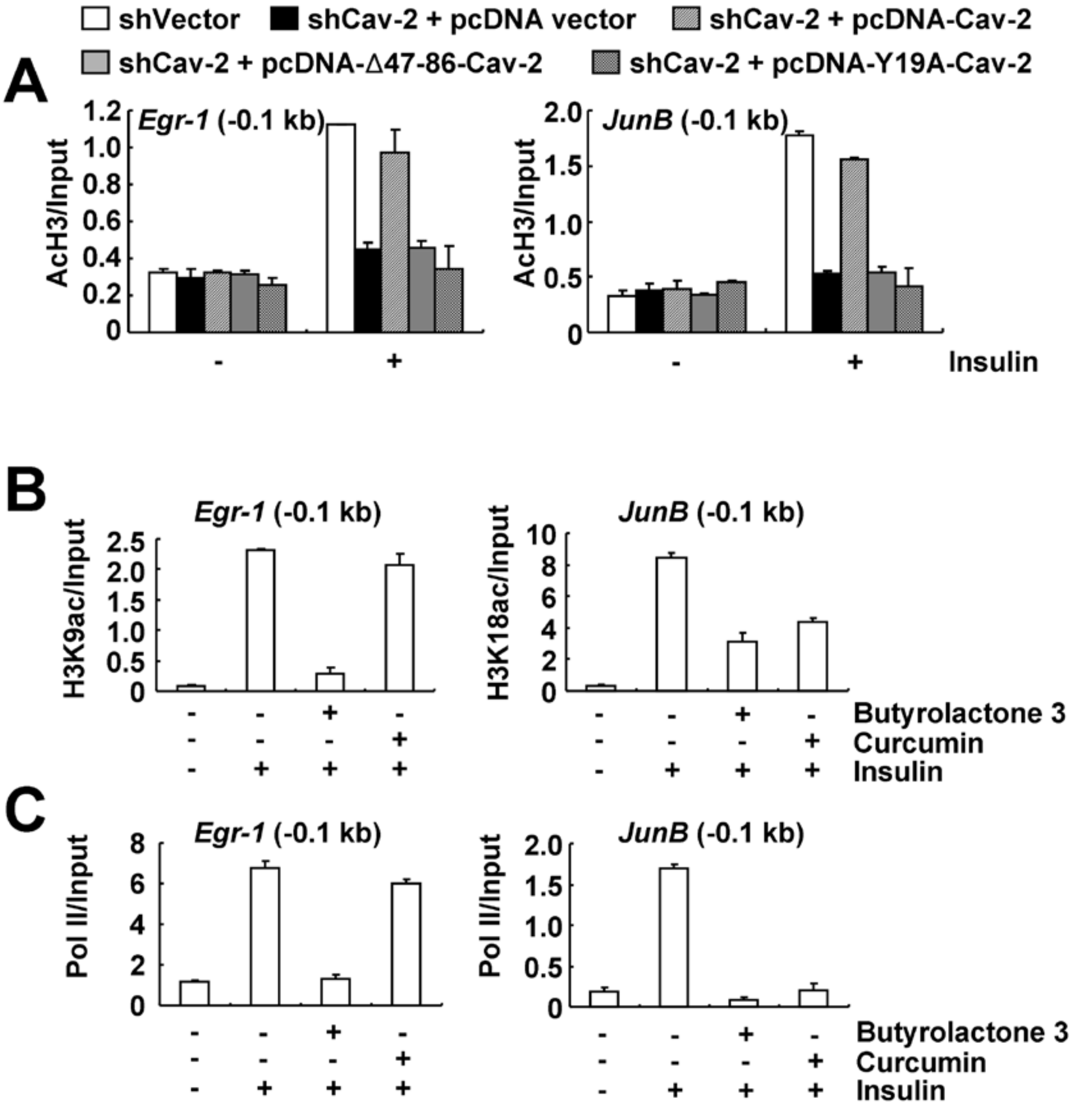

Supplement: SUPPLEMENTARY DATA [file supp_gkv181_nar-03733-x-2014-File008.pdf]
